# Factors That Facilitate Discussion and Documentation of End-of-Life Care among Community-Dwelling Older Adults: A Cross-Sectional Study

**DOI:** 10.3390/ijerph19074273

**Published:** 2022-04-02

**Authors:** Tomoyuki Ishibashi, Kana Kazawa, Yasmin Jahan, Michiko Moriyama

**Affiliations:** 1 Division of Nursing Science, Graduate School of Biomedical and Health Sciences, Hiroshima University, Hiroshima 734-8553, Japan; tatibanasora9249@yahoo.co.jp (T.I.); dr.yasminjahan@gmail.com (Y.J.); morimich@hiroshima-u.ac.jp (M.M.); 2 Department of Medicine for Integrated Approach to Social Inclusion, Graduate School of Biomedical and Health Sciences, Hiroshima University, Hiroshima 734-8553, Japan

**Keywords:** older adults, end-of-life care, decision-making

## Abstract

We aimed to clarify the regional cultural characteristics in areas with different death rates at home, and to identify factors that influence the discussion and documentation of end-of-life care (EOLC) among community-dwelling older adults. This study was a cross-sectional study using a self-administered questionnaire survey, and participants were Japanese older adults. A chi-square test and multiple regression analysis were conducted. Among the 227 respondents, 143 were analyzed. There were no statistical differences by area. Participants who had intentions to discuss EOLC tended to discuss EOLC with their families and family doctors and tended to create documents to show their wills on EOLC (*p* < 0.05). The following factors that influence the intentions to discuss EOLC were extracted: experience in providing EOLC; information on EOLC; having religious and spiritual beliefs, and not avoiding the subject of death as part of beliefs related to life and death. These results indicate that beliefs and intentions regarding EOLC may be similar across Japan. Moreover, our findings suggest that to increase the interest of older adults on EOLC, it is important to provide opportunities for older adults to share and discuss information about EOLC with healthcare professionals and others who have experience providing EOLC.

## 1. Introduction

Aging populations in developed countries have increased dramatically. Japan has an advanced aging population; as of 2021, 28.9% of people were aged 65 years and above [1,2]. Moreover, in Japan, the rapid decrease in the birth rate has led to a decrease in the working-age population who support the lives of older adults [2]. In particular, end-of-life care (EOLC) for older adults has become a compelling issue for the country. In response to this situation, the government has transformed “hospital-centered medical care” to “community-oriented medical care”, where medical care, long-term care, and welfare are integrated [3]. Meanwhile, advancements in medicine and increasing care options are forcing older adults and their families to make critical decisions about medical care, including EOLC. A survey of Japanese older adults found that 58.8% of respondents wished to spend the end of their lives at home in 2020 [4]. However, only 13.6% of those died at home in 2019 [5]. Thus, there is a significant gap between where people want to die and where they actually die.

Causes of such a gap, which pose obstacles to Japanese older adults in the discussion and documentation of EOLC (leading to preferred choice and place of death), include the following; strong tendencies to leave decisions to their doctors [6,7]; lack of communication on end-of-life plans between older adults, their families, and healthcare professions; lack of legal frameworks for preparing end-of-life plans; and lack of knowledge of advanced care planning (ACP) among relatives [8]. ACP is a strategy used to communicate and make future healthcare plans, which include EOLC. This form of planning increases “the ability to enable individuals to define goals and preferences for future medical treatment and care, to discuss these goals and preferences with family and healthcare providers, and to record and review these preferences if appropriate” [9]. Moreover, it is important that the patients and their proxies share personal values, life goals, and preferences regarding future medical care, so that the proxies can make decisions for the patients when the patients can no longer make decisions themselves [10]. Studies have found that decision-making processes can improve patient autonomy; alleviate decisional conflicts; improve communication between the patients, their families, and health professionals; increase preference-concordant care, and facilitate patients living out the end of their lives in their preferred settings [11,12,13,14,15]. The above findings suggest that it is of utmost importance for Japanese citizens to discuss and document their own EOLC because it may lead to improvements in the quality of life of older adults and their families, quality EOLC, and policy improvement.

The following factors were found to influence the discussion and documentation of EOLC: (1) a person’s individual factors, namely age, gender, intentions, religious and spiritual beliefs, beliefs about life and death, physical functioning and disease progression, and experience in providing EOLC; and (2) environmental factors, namely local health/community services (e.g., resource availability, relationship, and improvement and enhancement of home care), and caregivers, including family members (e.g., availability of caregivers, relationship, will, and coping skills) [16,17,18,19,20,21]. However, to the best of our knowledge, no research has been done on the backgrounds of such regional cultural differences. Moreover, few studies have conducted exploratory analyses on the environmental factors that directly or indirectly affect discussing and documenting EOLC, such as living wills among older adults in local communities.

This study aimed to clarify whether there are regional cultural characteristics in areas with different death rates at home, and to identify factors that influence discussions and documentation of EOLC among older adults. It is expected that the findings of this study will help older adults (and their families) realize their desired EOLC.

## 2. The Conceptual Framework of This Study

**Figure 1:**
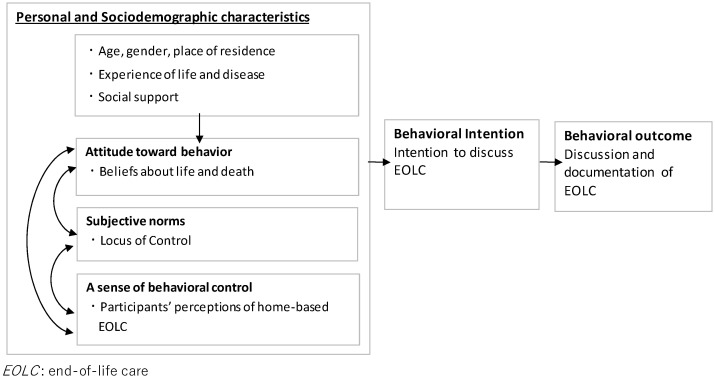
Conceptual framework of this study.

This study employed Ajzen’s theory of planned behavior [22] in its investigation of the intentions and behaviors related to the discussion and documentation of EOLC. According to Ajzen’s theory, in order for an individual to engage in a certain behavior, the individual needs to have an intention to engage in that behavior. The theory also states that intentions to engage in a certain behavior can be affected by subjective norms (social and interpersonal factors), attitude towards the behavior (beliefs about the behavior), and a sense of behavioral control (ease of the behavior). Based on Ajzen’s theory and preceding studies, this study considered “discussion and documentation of EOLC” as the behavior outcome, and “intentions to discuss EOLC” as the behavioral intention. The study also considered “beliefs about life and death”, which is an attitude towards life and death, as an attitude towards the behavior, and “the locus of control” as the subjective norm (Figure 1). Moreover, based on preceding studies, this study assumed that home-based EOLC can be facilitated when support and understanding from one’s family and community are available. Hence, this study equated “views held by family and the community on home-based EOLC” with a sense of behavioral control.

## 3. Methods

### 3.1. Study Design and Recruitment

A comparative cross-sectional study using a self-administered questionnaire was conducted. Participants were recruited among local older adults with the help of the head of the neighborhood association. The study was conducted from September to December 2018. The researchers attended community meetings and questionnaire forms were distributed to participants after they received written and oral information about the study. Participants completed the questionnaire at the recruitment site and placed completed forms in the collection box, which was placed at the site. Their responses were anonymous. Participants were deemed to have given consent to participate in the study upon submission of the questionnaire form.

### 3.2. Participants

In Japan, the death rates at home vary among regions. Therefore, in order to investigate the regional cultural differences, this study was conducted in two areas. One was an urban area where medical institutions are concentrated (Minami-ku, Hiroshima City, Hiroshima; Hiroshima) and the other was a mountainous area where few medical institutions are located (Nagi Town, Katsuta-gun, Okayama; Nagi).

The proportion of people aged 65 years or older in Hiroshima was 23.0% in 2015 (national mean: 26.0%). It has a larger number of hospitals, clinics, and hospital beds than the national means [23]. The rate of home-based EOLC for the city was 22.6% in 2018 (national mean: 20.7%) [24]. On the contrary, the proportion of people aged 65 years or older in Nagi was 33.0% in 2015, which was higher than the national mean and Nagi has only two clinics (JMA, 2020). However, it has implemented projects that facilitate integrated care and home-based care. Nagi’s rate of home-based EOLC in 2018 was 48.1%, which was significantly higher than the national mean [24].

Eligibility criteria were: living at home, being aged 65 years or older, and being able to understand and complete the questionnaire items by themselves.

The sample size was calculated. To detect associations between regions and measures, a two-sided α = 0.05, β = 0.2 and effect size 0.30 were used, and 88 cases were identified as the target sample size.

In the present study, the questionnaire was distributed to 227 older adults, all of whom responded. Of these, 29 older adults did not fulfill the eligibility criteria, and 55 did not provide complete responses for each question. After excluding these respondents, data from the remaining 143 participants were analyzed (valid response rate: 63.0%). 

Table 1 shows the basic attributes and disease factor of the groups that did not complete the questionnaire and those that did. Compared to the group that completed the questionnaire, the group that did not complete it tended to be slightly older. Regarding the characteristics of the 143 participants, the gender breakdown of the total eligible participants was 36 men (25.2%) and 107 women (74.8%). The mean (± SD) age was 76.6 ± 5.8 years.

### 3.3. Data Collection and Measurement

All data for the evaluation were collected from questionnaire forms completed by the participants.

#### 3.3.1. Personal and Sociodemographic Characteristics

- Basic attributes: age, gender, place of residence.
- Disease and life experience: participants were asked if they had any diseases, religious and spiritual beliefs (Yes: 1, No: 0).
- Social support: participants were asked if they could receive support from health professionals, family, or friends (Yes: 1, No: 0).
- Attitude towards behavior: a scale for beliefs about life and death was used to measure attitude towards the behavior [25]. The scale measures values and attitude towards death. It consists of seven subscales (views on life after death, fear and anxiety for death, death as liberation, avoidance of death, a sense of purpose for life, interest in death, and perceived life expectancy) and 27 items. Participants answered the questions on a seven-point ranging scale. The higher the score, the stronger the belief. Scores for the seven subscales were summed to obtain total scores, which were used in evaluating the characteristics of participants’ beliefs about life and death. Cronbach’s alpha in the present study is 0.751.
- A sense of behavioral control: two items that the researchers created were used to measure participants’ perceptions of home-based EOLC: whether or not participants thought that home-based EOLC increased the burden of their families and whether or not participants thought that many people in their communities considered home-based EOLC to be natural.
- Subjective norms: the Japanese version of the Health Locus of Control (LOC) scale was used to develop questions for a sense of behavioral control [26]. The scale effectively reflected Japanese cultural views. Instead of a dichotomous structure of internal and external factors, the Japanese version of the Health LOC Scale consists of 25 question items and the following five subscales: self, family, professionals, coincidence, and supernaturalism. Participants answered the questions on a six-point scale ranging in descending order. The higher the score, the stronger the LOC. Scores for the five subscales were summed to obtain total scores, which were used in evaluating the characteristics of participants’ LOC (Yes: 1, No: 0). Cronbach’s alpha in the present study is 0.678.

#### 3.3.2. Behavioral Intention

The behavioral intention was measured with one question on whether or not participants had intentions to discuss EOLC, i.e., leading to preferred choice and place of death (Yes: 1, No: 0).

#### 3.3.3. Behavioral Outcomes

Behavioral outcomes were measured with one question each, regarding the participants’ experiences in discussing their own EOLC with their families and doctors, and whether or not they had created documents on EOLC, i.e., leading to preferred choice and place of death (Yes: 1, No: 0).

### 3.4. Statistical Analysis

Descriptive statistics were used to analyze personal and sociodemographic characteristics, behavioral intentions, and behavioral outcomes. Cronbach’s α coefficients to confirm the reliability of each scale and correlations were calculated.

First, we compared measures of two regions with different death rates at home. Next, the relationship between behavioral intention and behavioral outcome was analyzed. Moreover, in order to clarify personal and sociodemographic characteristics that directly affect behavioral intentions in line with the given framework, a backward likelihood ratio binary logistic regression analysis was performed using basic attributes, attitude toward behaviors, subjective norms, and a sense of behavioral control as the independent variables after confirming that the inter-variable correlation coefficient between the variables was |r| < 0.8. Variable reduction was determined by the *p*-value (*p* > 0.10). For statistical analysis, the statistical software SPSS version 25 (IBM, Armonk, NY, USA) was used, with the significance level set at under 5%.

This research was reported according to the Strengthening the Reporting of Observational Studies in Epidemiology (STROBE) statement [27].

### 3.5. Ethical Consideration

The study protocol was approved by Ethics Committee for Epidemiology of Hiroshima University (Hiroshima, Japan). We considered it as consent when the participant submitted the questionnaire form. This study was performed in accordance with the Declaration of Helsinki and the Ethical Guidelines of the Ministry of Health, Labour, and Welfare of Japan.

## 4. Results

### 4.1. Comparison of the Two Regional Areas

Table 2 shows a comparison between Hiroshima and Nagi. The two areas where there is a difference in death rates at home, the following numbers of participants answered that they had the intention to discuss EOLC: 46 (57.5%) in Hiroshima and 36 (59.0%) in Nagi. Regarding behavioral outcome, a slightly higher proportion of participants in Hiroshima answered that they discussed EOLC with their families compared with those in Nagi. No notable difference was observed between the two areas in the proportion of participants who indicated that they had discussed EOLC with their family doctors. A slightly higher proportion of participants in Nagi answered that they had created documents on EOLC compared with those in Hiroshima. It is worth noting that in Nagi, 12 participants (21.4%) answered that many people in their community recognized home-based EOLC as a natural practice, whereas 9 in Hiroshima (11.7%) answered the same. Additionally, 42 participants in Hiroshima (56.0%) considered it difficult to receive home-based EOLC, whereas 28 in Nagi (50.9%) answered the same. No significant differences were observed between the two areas.

### 4.2. Relationship between Behavioral Intentions and Behavioral Outcomes (All Participants)

Table 3 shows data on the relationship between behavior intention and behavior outcome. The participants who had intentions to discuss EOLC were more likely to discuss EOLC with their families and family doctors and to create documents on EOLC (*p* < 0.05).

### 4.3. Personal and Sociodemographic Factors That Affect Behavioral Intention (All Participants)

To identify the factors that directly affect participants’ behavioral intentions (intention to discuss EOLC), a logistic regression analysis was performed with behavioral intention as the dependent variable, and beliefs about life and death, LOC, subjective norms, and other personal and sociodemographic factors as independent variables (Table 4). The strongest direct impact on participants’ behavioral intentions was experience in providing EOLC, followed by information on EOLC, having religious and spiritual beliefs, and avoidance of death, which was one of the subscales of the scale for beliefs about life and death. Among these four significant indicators, only avoidance of death had a negative impact on participants’ behavioral intentions, suggesting that those who do not try to avoid death are more likely to consider their own EOLC.

## 5. Discussion

This study aimed to clarify the regional cultural characteristics in areas with different death rates at home, and to identify factors that influence the discussion and documentation of EOLC, which could lead to preferred choice and place of death among community-dwelling older adults. The results showed that among all of the analyzed participants, 58.2% had the intention to discuss EOLC, 46.9% discussed EOLC with their families, and 8.4% had created documents on EOLC. A national Japanese survey in 2018 found that 46.6% of respondents aged 60 years or older had discussed EOLC with their families and 13.6% of the respondents had created documents on EOLC [5]. A comparison of the response rates between this study and the national survey indicated the percentage of participants in this study who had created documents on EOLC was slightly lower.

### 5.1. An Overview of Study Participants and a Comparison between Two Regional Areas with Different Death Rates at Home

This study examined two areas with different death rates at home, namely Hiroshima (an urban area) and Nagi (a rural area), with the aim of clarifying whether there were regional cultural characteristics. There were no differences between two areas. The results may indicate that beliefs and intentions regarding EOLC are similar across Japan. However, there are several possible factors that may contribute to the higher death rate at home in Nagi. In this study, compared with the participants in Hiroshima, a slightly higher proportion of participants in Nagi answered that many people in their community recognized home-based EOLC as a natural practice. Since 2006, family-based primary care has been practiced in Nagi, which has led to the increased availability of 24-h health services and home-based EOLC [28]. This result may indicate that the potential environmental factors such as local structural and institutional supports, create better EOLC rates than the residents’ intentions to discuss or document EOLC.

### 5.2. Relationship between the Intention to Discuss EOLC and Behavioral Outcome

The participants who had intentions to discuss EOLC were more likely to discuss EOLC with their families and family doctors and to create documents on EOLC. The framework of this study was based on Ajzen’s theory of planned behavior. The results suggested that, in order to promote discussion and documentation of EOLC among the older adults, it is necessary to raise awareness of EOLC.

Meanwhile, a large gap was found between the proportion of participants who discussed EOLC with their families and the proportions of participants who discussed EOLC with family doctors, and who created documents for EOLC. When supporting patients in making decisions on their chosen ways of ending life/EOLC, it is important that healthcare providers explain the care plans to the patients and provide options for medical treatment and care. It is also required that the patient, his/her family, and other relatives, such as healthcare professionals, share the patient’s intentions, respect the patient’s autonomy, and document discussions in writing [29,30]. If the patient is unable to make a decision, a document of EOLC may help avoid conflict and mitigate ethical conflicts among the patient’s family, healthcare professionals, and others. Moreover, the process of EOLC should ideally be discussed often because the patient may change his or her mind regarding EOLC plans, depending on the time and situation.

### 5.3. Factors That Affect the Intention to Discuss EOLC

To identify factors that directly affect participants’ behavioral intentions, a logistic regression analysis was performed. The strongest direct impact on participants’ behavioral intentions was the experience in providing EOLC, followed by having information on EOLC, religious and spiritual beliefs, and avoidance of death. These factors are consistent with findings from preceding studies [6,20,31,32].

In particular, an investigational study in Japan reported that one’s experience in providing EOLC, which was found to have the strongest impact on behavioral intentions in this study, leads a caregiver to proactively contemplate death [32,33]. The experience in providing EOLC may be an opportunity for the caregiver to consider EOLC for him/herself. In order to support patient- and family-centered decision-making, it may be necessary to provide them with interventions that focus on achieving a consensus among them on their preferred end-of-life approaches. Such interventions may include providing older adults with opportunities to share their experiences of providing EOLC with others with similar experience and health professionals or helping older adults notice the positive aspects of involving their families in EOLC.

In this study, information on EOLC was found to have the second strongest impact on participants’ behavioral intentions. In a previous study [7], the majority of respondents indicated that they could not image where they wanted to receive EOLC once they needed it. Few respondents indicated having a preferred location. The reasons why older adults cannot think of their preferred situations and locations of receiving EOLC may include the advancements in medicine, many options, and low information literacy. In particular, older adults may find it difficult to understand information provided by health professionals at once. Structured programs were reported to improve older adults’ understanding and awareness of ACP and advanced directives [34,35]. Hence, accurate communication information regarding EOLC based on the ACP process between older adults and healthcare professionals can help them recognize EOLC as an issue relevant to themselves.

### 5.4. Study Limitations and Significance

This study has a few limitations. First, because this was a descriptive study, it could not infer causal relationships. Second, this study involved residents of two areas, and the results may not be generalizable. However, to the best of our knowledge, no previous study has comprehensively analyzed and reported the influential factors of EOLC through research that compares the areas with different death rates at home. Third, this study met the required sample size; however, the result did not show regional differences. This result may indicate that the actual effect size was smaller than the assumed effect size. The valid response may affect the results. Therefore, increasing fields and participants will enhance our understanding of this issue.

## 6. Conclusions

This study aimed to clarify whether there are regional cultural characteristics in areas with different death rates at home, and to identify factors that influence the discussion and documentation of EOLC among older adults. The results indicate that there may be no differences in older adults’ beliefs or intentions regarding the discussion and documentation of EOLC by region. Moreover, the results revealed that older adults who intend to discuss EOLC are likely to discuss EOLC with their families and family doctors, as well as create documents on EOLC. Experience in providing EOLC, information on EOLC, having religious and spiritual beliefs, and not avoiding the subject of death as part of one’s beliefs related to life and death, were extracted as factors that directly influence one’s intention to discuss EOLC. 

The above findings suggest that providing older adults with opportunities to share and discuss their experiences of providing EOLC, and information on EOLC with healthcare professionals and others who have experience in providing EOLC, may help them consider EOLC for themselves. This may also help increase their interests in EOLC and lead to more positive perceptions on EOLC, for themselves and their families.

## Figures and Tables

**Table 1:** Comparison between people who did not complete the questionnaire and people who completed the questionnaire.

Factor	People Who Did Not Complete the Questionnaire						People Who Completed the Questionnaire					
	Hiroshima			Nagi			Hiroshima			Nagi		
	All	Applicable Participants		All	Applicable Participants		All	Applicable Participants		All	Applicable Participants	
	n	n	%	n	n	%	n	n	%	n	n	%
Gender: Male	29	8	27.6	26	5	19.2	82	21	25.6	61	15	24.6
Age: years (mean ± SD)	29	80.8	±6.7	26	82.0	±6.8	82	77.0	±5.7	61	76.0	±6.0
Experience of life-threatening disease: Yes	16	2	12.5	19	5	26.3	80	24	30	61	16	26.2

**Table 2:** Comparisons between Hiroshima and Nagi.

	**Hiroshima**			**Nagi**			**Comparison of Areas**	
	**Analyzed Participants**	**Applicable Participants**		**Analyzed Participants**	**Applicable Participants**			
	**n**	**n**	**%**	**n**	**n**	**%**	***p*-Value**	
Gender: Male	82	21	25.6	61	15	24.6	0.889	^a^
Age: years (mean ± SD)	82	77.0	±5.7	61	76.0	±6.0	0.290	^c^
Behavioral Intention	80	46	57.5	61	36	59.0	0.856	^a^
Intention to discuss EOLC: Yes								
Behavioral outcome								
Discussed EOLC with their family: Yes	80	40	50.0	61	27	44.3	0.723	^a^
Discussed EOLC with family doctor: Yes	82	9	11.0	60	6	10.0	0.540	^b^
Created a document on EOLC: Yes	81	6	7.4	59	6	10.2	0.389	^b^
Social Support								
Having a family doctor: Yes	81	75	92.6	61	53	86.9	0.259	^a^
Support by health professionals: Yes	79	22	27.8	60	20	33.3	0.485	^a^
Support by family: Yes	82	40	48.8	60	30	50.0	0.886	^a^
Support by friend: Yes	79	30	38.0	59	17	28.8	0.261	^a^
Experience of life and disease								
Experience of a life-threatening disease: Yes	80	24	30.0	61	16	26.2	0.623	^a^
With an illness undergoing treatment: Yes	81	51	63.0	60	45	75.0	0.130	^a^
Having religious and spiritual belief: Yes	81	29	35.8	61	16	26.2	0.225	^a^
Experience in providing EOLC: Yes	80	64	80.0	60	51	85.0	0.445	^a^
Having media information on EOLC: Yes	82	60	73.2	61	49	80.3	0.253	^a^
**Factor**	**Hiroshima**			**Nagi**			**Comparison of Areas**	
	**All**	**Applicable Participants**		**All**	**Applicable Participants**			
	**n**	**n**	**%**	**n**	**n**	**%**	***p*** **-Value**	
Attitude towards behavior (Scale for beliefs about life and death): mean ± SD								
Views on life after death	72	13.4	±5.4	54	13.1	±5.8	0.805	^c^
Fear and anxiety for death	72	14.4	±5.8	54	14.7	±6.5	0.800	^c^
Death as liberation	72	14.8	±6.4	54	14.5	±7.2	0.769	^c^
Avoidance of death	72	13.3	±5.2	54	13.1	±6.4	0.840	^c^
A sense of purpose for life	72	15.5	±4.6	54	14.2	±6.0	0.184	^c^
Interest in death	72	13.9	±5.6	54	14.6	±6.1	0.525	^c^
Perceived life expectancy	72	12.4	±4.6	54	11.6	±5.1	0.362	^c^
A sense of behavioral control								
Many people in my community recognize home-based EOLC as natural practice: Yes	77	9	11.7	56	12	21.4	0.152	^b^
Home-based EOLC is burden for my family: Yes	75	42	56.0	55	28	50.9	0.565	^b^
Subjective norms (LOC score): mean ± SD								
Supernaturalism	77	14.6	±3.9	55	13.8	±4.3	0.329	^c^
Self	77	24.4	±3.3	55	23.8	±3.1	0.297	^c^
Coincidence	77	16.9	±4.1	55	17.0	±5.1	0.849	^c^
Family	77	22.4	±3.8	55	22.3	±3.3	0.771	^c^
Professionals	77	20.0	±3.4	55	19.5	±4.4	0.429	^c^

Note. EOLC = end-of-life care; LOC = locus of control. Cronbach’s α for LOC score, scale for beliefs about life and death were 0.678, 0.751, respectively. ^a^ Pearson’s χ2 test; ^b^ Fisher’s exact test; ^c^ *t*-test.

**Table 3:** The relationship between behavioral intention and behavioral outcome.

Behavior Outcome	Analyzed Participants		Behavior Intention				*p*-Value	
			Intention to Discuss EOLC					
			Yes		No			
			n	%	n	%		
Discussed EOLC with their family	139	Yes	60	43.2	6	4.3	<0.001	***
		No	22	15.8	51	36.7		
Discussed EOLC with family doctor	140	Yes	14	10.0	1	0.7	0.004	**
		No	68	48.6	57	40.7		
Created a document on EOLC	138	Yes	11	8.0	1	0.7	0.015	*
		No	70	50.7	56	40.6		

Note. EOLC = end-of-life care.. Fisher’s exact test. * *p* < 0.05; ** *p* < 0.01; *** *p* < 0.001.

**Table 4:** Personal and sociodemographic factors that affect behavioral intention (intention to discuss EOLC).

Factor	B	OR	95%CI		*p*-Value	
			Lower	Upper		
Having a family doctor	1.660	5.259	0.717	38.568	0.103	
Having religious and spiritual belief	1.429	4.175	1.098	15.877	0.036	*
Experience in providing EOLC	2.369	10.682	2.299	49.636	0.003	**
Having media information on EOLC	1.882	6.567	1.593	27.074	0.009	**
Beliefs about life and death						
Views on life after death	−0.125	0.883	0.773	1.008	0.065	
Avoidance of death	−0.191	0.526	0.728	0.938	0.003	**
Interest in death	0.102	1.107	0.995	1.233	0.063	
LOC						
Supernaturalism	0.174	1.190	0.988	1.433	0.067	
Professionals	−0.150	0.861	0.719	1.233	0.102	

Note. B = 101; EOLC = end-of-life care; LOC = locus of control. The discriminant predictive value 56.4%. Hosmer–Lemeshow test *p* = 0.598. * *p* < 0.05; ** *p* < 0.01.

## Data Availability

All data that support the findings of this study are available from the corresponding author upon reasonable request.

## References

[1] Organization for Economic Co-Operation and Development (2021). OECD. Stat. https://stats.oecd.org/#.

[2] Ministry of Internal Affairs and Communications (2020). Population and Households. Statistics in Japan 2020. http://www.stat.go.jp/data/nihon/02.html.

[3] Arai H., Ouchi Y., Toba K., Endo T., Shimokado K., Tsubota K., Matsuo S., Mori H., Yumura W., Yokode M. (2015). Japan as the front-runner of super-aged societies: Perspectives from medicine and medical care in Japan. Geriatr. Gerontol. Int..

[4] The Nippon Foundation (2020). A Survey on Sense of End-of-Life-Care of the Older Adults. https://www.nippon-foundation.or.jp/who/news/pr/2021/20210329-55543.html.

[5] Statistics Bureau of Japan (2019). A Survey on Place of Death. The Portal Site of Official Statistics of Japan (e-Stat). https://www.e-stat.go.jp/dbview?sid=0003411652.

[6] Matsui M., Moriyama M. (2004). The interest and related factors concerning introductory educational activities as to terminal care among the elderly. J. Jpn. Assoc. Bioeth..

[7] Hirakawa Y., Masuda Y., Kazuya M., Lguchi A., Uemura K. (2006). Older person’s preferences for site of end-of-life care and living will. Hosp. Home Care.

[8] Takeshita Y., Ikeda M., Sone S., Moriyama M. (2015). The Effect of Educational Intervention regarding Advance Care Planning for Advance Directives. Health.

[9] Rietjens J.A.C., Sudore R.L., Connolly M., van Delden J.J., Drickamer M.A., Droger M., van der Heide A., Heyland D.K., Houttekier D., Janssen D.J.A. (2017). European Association for Palliative Care. Definition and recommendations for advance care planning: An international consensus supported by the European Association for Palliative Care. Lancet Oncol..

[10] Sudore R.L., Lum H.D., You J.J., Hanson L.C., Meier D.E., Pantilat S.Z., Matlock D.D., Rietjens J.A.C., Korfage I.J., Ritchie C.S. (2017). Defining Advance Care Planning for Adults: A Consensus Definition from a Multidisciplinary Delphi Panel. J. Pain Symptom Manag..

[11] Fried T.R., O’Leary J.R. (2008). Using the experiences of bereaved caregivers to inform patient- and caregiver-centered advance care planning. J. Gen. Intern. Med..

[12] Landmark A.M.D., Gulbrandsen P., Svennevig J. (2015). Whose decision? Negotiating epistemic and deontic rights in medical treatment decisions. J. Pragmat..

[13] Yamamoto S., Arao H., Masutani E., Aoki M., Kishino M., Morita T., Shima Y., Kizawa Y., Tsuneto S., Aoyama M. (2017). Decision Making Regarding the Place of End-of-Life Cancer Care: The Burden on Bereaved Families and Related Factors. J. Pain Symptom Manag..

[14] Jimenez G., Tan W.S., Virk A.K., Low C.K., Car J., Ho A.H.Y. (2018). Overview of Systematic Reviews of Advance Care Planning: Summary of Evidence and Global Lessons. J. Pain Symptom Manag..

[15] Fahner J.C., Beunders A.J.M., van der Heide A., Rietjens J.A.C., Vanderschuren M.M., van Delden J.J.M., Kars M.C. (2019). Interventions Guiding Advance Care Planning Conversations: A Systematic Review. J. Am. Med. Dir. Assoc..

[16] Gomes B., Higginson I.J. (2006). Factors influencing death at home in terminally ill patients with cancer: Systematic review. BMJ.

[17] Araki A., Horiuchi F., Asano Y. (2010). The Wishes of Elderly People Regarding Preparations for the End of Life and Related Factors. J. Jpn. Acad. Home Health Care.

[18] Yamagishi A., Morita T., Miyashita M., Yoshida S., Akizuki N., Shirahige Y., Akiyama M., Eguchi K. (2012). Preferred place of care and place of death of the general public and cancer patients in Japan. Support. Care Cancer.

[19] Kinoshita H., Maeda I., Morita T., Miyashita M., Yamagishi A., Shirahige Y., Takebayashi T., Yamaguchi T., Igarashi A., Eguchi K. (2015). Place of death and the differences in patient quality of death and dying and caregiver burden. J. Clin. Oncol..

[20] Shimada C., Nakazato K., Arai K., Aita K., Shimizu T., Tsurukawa M., Ishizaki T., Takahashi R. (2015). Communication with important others regarding their preferences for end-of-life care. Jpn. J. Geriatr..

[21] Costa V., Earle C.C., Esplen M.J., Fowler R., Goldman R., Grossman D., Levin L., Manuel D.G., Sharkey S., Tanuseputro P. (2016). The determinants of home and nursing home death: A systematic review and meta-analysis. BMC Palliat. Care.

[22] Ajzen I. (2012). The Theory of Planned Behavior. Handbook of Theories of Social Psychology.

[23] Japan Medical Association (2020). Japan Medical Analysis Platform. http://jmap.jp/.

[24] Ministry of Health, Labour and Welfare (2018). Data for Home Health Care by Region. https://www.mhlw.go.jp/stf/seisakunitsuite/bunya/0000061944.html.

[25] Hirai K., Sakaguchi Y., Abe K., Morikawa Y., Kashiwagi T. (2000). The study of death attitude: Construction and validation of the Death Attitude Inventory. Shi. No Rinsyo.

[26] Horike Y. (1991). A Japanese version of the Health Locus of Control Scales. Kenkou Shinrigaku Kenkyu..

[27] von Elm E., Altman D.G., Egger M., Pocock S.J., Gøtzsche P.C., Vandenbroucke J.P., STROBE Initiative (2008). The Strengthening the Reporting of Observational Studies in Epidemiology (STROBE) statement: Guidelines for reporting observational studies. J. Clin. Epidemiol..

[28] Matsushita A. (2019). Fostering primary care nurse and pharmacist who will play a new role in the community. Acad. Stud. J. Health Care Soc..

[29] Truglio-Londrigan M., Slyer J.T. (2018). Shared Decision-Making for Nursing Practice: An Integrative Review. Open Nurs. J..

[30] Bomhof-Roordink H., Gartner F.R., Stiggelbout A.M., Pieterse A.H. (2019). Key components of shared decision making models: A systematic review. BMJ Open.

[31] Tomoi K. (2019). Preferred Place of Death and Death at Home Based on a Survey of Local Residents. Hosp. Home Care.

[32] Inagaki A., Takano J., Noguchi-Watanabe M., Yamamoto-Mitani N. (2020). Exploring Factors of Advance Care Planning Practice among Community-dwelling Independent Older Adults: A Cross-sectional Study. J. Jpn. Acad. Nurs. Sci..

[33] Kunugi N., Ono M. (2018). Examination of Factors Related to Older People’s Views of Life and Death. Hosp. Home Care.

[34] Wu C.-H., Perng S.-J., Shi C.-K., Lai H.-L. (2020). Advance Care Planning and Advance Directives: A Multimedia Education Program in Community-Dwelling Older Adults. J. Appl. Gerontol..

[35] Lin L.H., Cheng H.C., Chen Y.C., Chien L.Y. (2021). Effectiveness of a video-based advance care planning intervention in hospitalized elderly patients: A randomized controlled trial. Geriatr. Gerontol. Int..

